# Postoperative outcome and influencing factors of strabismus surgery in infants aged 1–6 years

**DOI:** 10.1007/s00417-024-06404-1

**Published:** 2024-02-16

**Authors:** Laetitia Hinterhuber, Sandra Rezar-Dreindl, Ursula Schmidt-Erfurth, Eva Stifter

**Affiliations:** Department of Ophthalmology and Optometry, Medical University of Vienna, Spitalgasse 23, 1090 Vienna, Austria

**Keywords:** Strabismus surgery, Infant, Influencing factors, Outcomes, Surgical success

## Abstract

**Purpose:**

To evaluate the postoperative outcome of strabismus surgery performed in children aged 1–6 years by investigating the change of the preoperative angle of deviation (AOD), elevation in adduction, best-corrected visual acuity (BCVA) and refractive error.

**Methods:**

Retrospective chart review of 62 children who received strabismus surgery between January 2018 and December 2021 at the Department of Ophthalmology and Optometry of the Medical University of Vienna. Age, sex, type of strabismus, AOD, BCVA, refractive error and visual acuity were evaluated with respect to the postoperative outcome.

**Results:**

Mean follow-up was 13.55 ± 11.38 months with a mean age of 3.94 ± 1.97 years (range: 1.0–6.0) at time of surgery. 74.19% of patients (*n* = 46) had isolated or combined esotropia, 12.90% (*n* = 8) had isolated or combined exotropia and 12.90% (*n* = 8) had isolated strabismus sursoadductorius. Mean preoperative AOD of 15.69 ± 16.91°/15.02 ± 14.88° (near/distance) decreased to 4.00 ± 9.18°/4.83 ± 7.32° (near/distance) at final follow-up (*p* < 0.001). BCVA improved from 0.26 ± 0.26/0.25 ± 0.23 (left/right) to 0.21 ± 0.25/0.20 ± 0.23 (left/right) (*p* = 0.038). There was no significant change regarding refractive error (*p* = 0.109) or elevation in adduction (*p* = 0.212). Success rate which was defined as a residual AOD of less than 10° was 74.19% (*n* = 46). In 3.23% (*n* = 2) retreatment was necessary.

**Conclusion:**

Strabismus surgery in infants was shown to have a satisfactory outcome with a low retreatment rate. Surgical success rate was not linked to age, sex, type of strabismus or the preoperative parameters AOD, refractive error and visual acuity in this study.

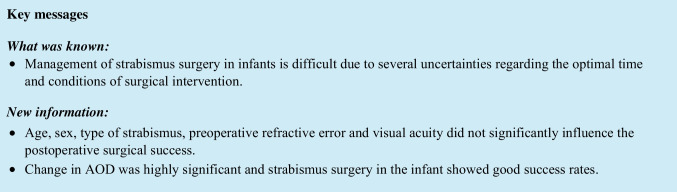

## Introduction

Strabismus is defined as a common ocular misalignment among children with a prevalence of 3–5% [1]. Surgical treatment remains a challenge since the surgical protocols are not standardized and there is still a lack of studies comparing surgical outcomes while considering the various influencing parameters [2].

The age group 1–6 years is particularly important because of the visual development and the impact of intervention on binocular function. Visual impairment during infancy and childhood can result in lifelong social and cognitive complications. The age of 1 to 2 years marks the growth of the optic nerves and the visual cortex. The fovea reaches maturity by the age of 4 and the rest of the visual system is fully developed by the age of 10 [3]. Amblyopia treatment yields the best results when performed before the age of 7, however the visual screening of children can pose problems regarding accuracy and testability [4]. To date, data concerning strabismus treatment in children are limited since the age group 1–6, especially if younger than three years, is often not included in studies [5].

Currently, there is no consensus on the optimal surgical approach for medium-angle strabismus with several different definitions of a successful surgical outcome and a wide age range of children undergoing the strabismus operation. In addition, many trials have focused on small-angle or large-angle strabismus while leaving out the moderate range between 10–20°.

This study was designed to evaluate the outcome of strabismus surgery in the age group 1–6 years and to assess the most important influencing factors in children undergoing a one-, two- or three-muscle surgery.

## Material and methods

A search of medical chart records at the Department of Ophthalmology and Optometry of the Medical University of Vienna was conducted to identify patients who underwent strabismus surgery between January 2018 and December 2021. Baseline characteristics as well as pre- and postoperative data were collected to identify which parameter significantly changed after surgery and how they influence the postoperative outcome.

Data consisted of the angle of deviation (AOD) obtained for near and distance, vertical deviation (VD) in proximity and distance, refractive error, best-corrected visual acuity (BCVA), binocular vision and mean duration until surgery or mean follow-up. Two follow-ups are compared with the first follow-up after one month or less and the second follow-up after at least three months after surgery.

Patients aged between 1–6 years that underwent their first strabismus surgery between January 2018 and December 2021 were included in this study. Exclusion criteria included patients outside the age range of 1–6 years, patients with a history of strabismus associated with systemic diseases, patients with history of strabismus because of brain tumors, patients with incomitant strabismus because of neurological diseases, patients with a history of penetrating or perforation eye trauma as well as patients with strabismus caused by severe head injury.

The analyzed demographic characteristics were age at time of surgery and sex, the functional outcome variables consisted of the mean AOD, BCVA and refractive error. The analyzed variables concerning the surgical procedure are the preoperative mean AOD (in degrees) as well as the type of surgery with the postoperative follow-up entailing the postoperative mean AOD, binocular vision function, BCVA and the mean refractive error.

Successful surgery outcome is defined as a postoperative angle of deviation of 10 degrees or less and is the main parameter to measure the success rate. All angles of deviation that were documented in prism diopters were converted in degrees by using the formula of *Lachenmayr *et al*.* [6] according to which 1.75 prism diopters correlates to a 1° deviation.

The angle of strabismus was measured in near and distance. Measurements were performed preoperatively, within one month and after at least three months following the surgery. AOD was measured in the primary position by the alternate prism cover test at fixed distances of 33 cm/6 m (near/distance) or by using the modified Krimsky’s test.

Visual acuity was determined through age-appropriate vision tests. The Cardiff-test was used for children under two years in this study. It is based on the Preferential Looking principle according to which a patterned target will rather be perceived than a plain stimulus with the same mean luminance [7]. Child-friendly symbols such as cars, fish or houses are depicted through black lines and used as stimuli. The lines are getting increasingly thinner to determine the child’s visual acuity [7–9]. Children over the age of two were examined through the Lea test. The vision sample chart consists of an apple, a pentagon, a square and a circle which the child has to name. The Lea test is known to be suitable for children of preschool age [8–10].

Stereopsis was determined using the Lang test, which examines stereoacuity by using the random-dot technique and a cylindrical grid.

All patients underwent complete ophthalmological examination before surgery and at every follow-up visit. Patients received full correction of the refractive error presurgically. Visual acuity was assessed after cycloplegic refraction.

All surgeries were performed under general anesthesia. A limbal approach and no adjustable sutures were used. In patients with esotropia, two-muscle surgery included medial rectus recession and lateral rectus plication and bilateral medial rectus recession and lateral rectus plication in three-muscle surgery. In patients with exotropia, two-muscle surgery included lateral rectus recession and medial rectus plication. Three-muscle surgery included bilateral lateral rectus recession and medial rectus plication. Surgery on the inferior oblique muscle was included in cases of overelevation in adduction. The method and dosage of surgery depended on the angle of strabismus. The surgical dosage is shown in Table 1.

**Table 1:** Surgical dosage table

Type of strabismus	Preoperative AOD/VD	Medial rectus recession (mm)	Lateral rectus recession (mm)
Esotropia	10	2,5	6,5
	20	3,5	8,5
	30	4,5	5
	40	4,5	9
Exotropia	10	3	4
	20	4,5	5,5
	30	5,5	7
	40	6,5	8
Strabismus sursoadductorius	5–20 VD	MOI Recession: 10-12mm with additional anteriorization (1-3mm)	

The study was approved by the ethics board of the Medical University of Vienna and adheres to the ethical principles for Medical Research of the Declaration of Helsinki. The statistical analysis was performed with Microsoft Excel and SPSS (IBM Statistics, Version 23). Continuous variables are presented as mean ± standard deviation or median and range, whereas categorical variables are presented as count and percentage. BCVA was transformed to logarithm of minimal angle of resolution (logMar) to create a linear scale for statistical analysis. A p-value ≤ 0.05 was considered as statistically significant. Final BCVA, spherical equivalent and mean AOD were compared pre- and postoperatively using Students t-test or Wilcoxon test. To evaluate the influencing factors of strabismus surgery a multivariate logistic regression was performed.

## Results

A total of 62 patients were included in this retrospective cohort study with a total follow-up of 13.55 ± 11.38 months. The mean age of the patients was 3.94 ± 1.97 years. The baseline characteristics are presented in Table 2.

**Table 2:** Baseline characteristics of children aged 1–6 years undergoing strabismus surgery. (*n* = 62)

		No (%)
Age at time of surgery	Mean age ± standard deviation (years)	3.94 ± 1.97
Gender	Male	32 (51.61)
	Female	30 (48.39)
Type of strabismus	Esotropia	23 (37.10)
	Combined Esotropia and Strabismus (Strab.) sursoadductorius	23 (37.10)
	Exotropia	5 (8.06)
	Combined Exotropia and Strab. sursoadductorius	3 (4.84)
	Isolated Strab. sursoadductorius	8 (12.90)

Out of the 46 esotropia patients, 32 underwent surgery on two extraocular muscles and 12 on three muscles. 87.50% of the exotropia patients were operated on two muscles and 12.50% on three muscles. The majority of patients diagnosed with isolated strabismus sursoadductorius had a unilateral surgery on the inferior rectus muscle (MOI) and one patient received bilateral surgery on the MOI.

Success rate, which was defined as a residual AOD of less than 10°, was 74.19% (*n* = 46). Tables 3, 4 and 5 describe the characteristics of patients with a postoperative AOD of ≤ 10° compared to those with a postoperative AOD of ≥ 10°. There is no general trend visible in the results regarding surgical success. Patients who achieved surgical success with either combined or isolated eso- or exotropia had a smaller preoperative AOD in comparison to those with a postoperative AOD ≥ 10° with a mean preoperative AOD of 19.19 ± 7.64 and 13.01 ± 23.40, respectively.

**Table 3:** Characteristics of patients with esotropia with postoperative AOD ≤ 10° and ≥ 10° (*n* = 46)

	Postoperative AOD ≤ 10°	Postoperative AOD ≥ 10°
Age at surgery	4.01 ± 1.32	2.97 ± 1.65
Gender (male:female)	21:13	5:7
Combined with Strab. sursoadductorius	18 (52.94%)	6 (50.00%)
Preoperative AOD	19.19 ± 7.64	24.02 ± 8.70
Preoperative refractive error	3.12 ± 1.95	2.22 ± 2.56
Preoperative visual acuity	0.22 ± 0.21	0.24 ± 0.21

**Table 4:** Characteristics of patients with exotropia with postoperative AOD ≤ 10° and ≥ 10° (*n* = 8)

	Postoperative AOD ≤ 10°	Postoperative AOD ≥ 10°
Age at surgery	3.83 ± 1.64	2.90 ± 0.00
Gender (male:female)	2:5	1:0
Combined with Strab. sursoadductorius	3 (42.86%)	0 (00.00%)
Preoperative AOD	13.01 ± 23.40	28.21 ± 0.00
Preoperative refractive error	0.21 ± 1.57	0.125 ± 0.00
Preoperative visual acuity	0.44 ± 0.24	0.20 ± 0.00

**Table 5:** Characteristics of patients with isolated strabismus sursoadductorius with postoperative AOD ≤ 10° and ≥ 10° (*n* = 8)

	Postoperative AOD ≤ 10°	Postoperative AOD ≥ 10°
Age at surgery	3.83 ± 0.31	4.03 ± 0.36
Gender (male:female)	2:4	1:1
Preoperative AOD	13.57 ± 0.61	12.00 ± 11.31
Preoperative refractive error	0.22 ± 4.95	3.19 ± 3.09
Preoperative visual acuity	0.20 ± 0.25	0.45 ± 0.28
Extraocular muscles (hypertropia:hypotropia)	4:2	0:2

### Angle of deviation

**Fig. 1:**
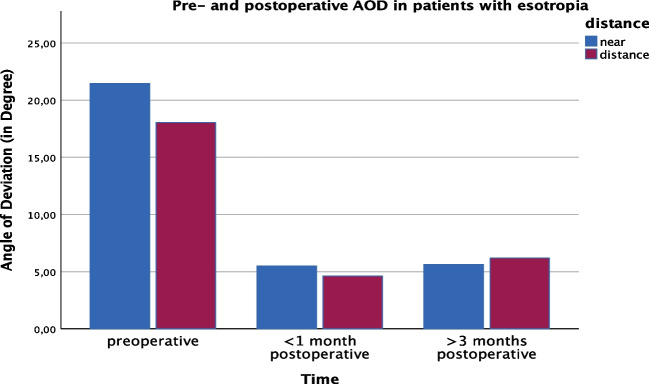
Pre- and postoperative AOD in patients with esotropia (*n* = 46)

The mean preoperative AOD in patients with esotropia was a mean of 21.46 ± 7.63/17.27 ± 8.26 (near/distance) respectively and improved to 5.50 ± 9.69/4.62 ± 7.71 at first follow up and to 5.62 ± 8.16/6.20 ± 5.30 at longterm follow up (*p* < 0.001) (Fig. 1).

**Fig. 2:**
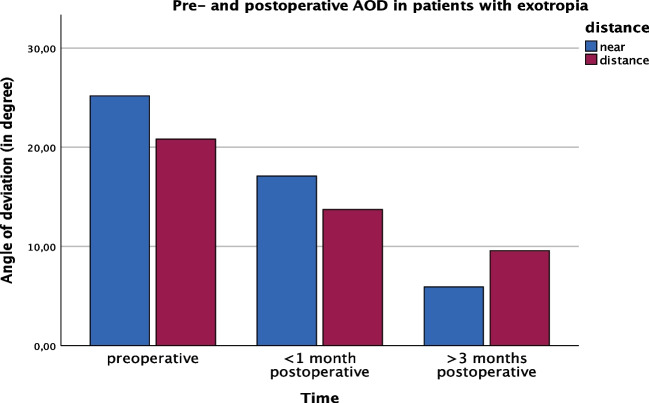
Pre- and postoperative AOD in patients with exotropia (*n* = 8)

The mean preoperative AOD in patients with exotropia was 15.36 ± 23.35/14.17 ± 16.95 (near/distance), 17.09 ± 16.17/13.71 ± 12.93 at first follow-up and 5.92 ± 5.12/0.43 ± 13.87 at longterm follow-up (*p* = 0.004) (Fig. 2).

**Fig. 3:**
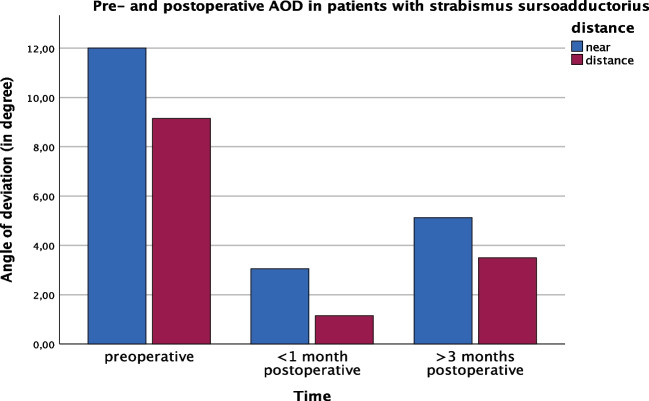
Pre- and postoperative AOD in patients with strabismus sursoadductorius (*n* = 8)

AOD in patients with isolated strabismus sursoadductorius improved from 12.00 ± 7.02/9.14 ± 11.31 (near/distance) preoperatively to 3.05 ± 3.67/1.14 ± 3.23 at first follow-up and 0.03 ± 10.54/0.71 ± 10.24 at longterm follow-up (*p* = 0.037) (Fig. 3).

### Elevation in adduction

27 patients had combined esotropia with overelevation in adduction. Preoperative vertical deviation in patients with combined esotropia and strabismus sursoadductorius decreased from 9.17 ± 3.72/7.27 ± 4.70 (near/distance) to 3.79 ± 1.86/3.43 ± 1.25 at first follow-up and 4.51 ± 4.64/4.07 ± 3.18 in the longterm follow-up (*p* = 0.943).

The vertical deviation in patients with combined exotropia and strabismus sursoadductorius changed from 3.81 ± 6.60/1.90 ± 3.30 (near/distance) presurgical to 0.95 ± 1.65/0.95 ± 1.65 at first follow-up and to 1.90 ± 1.65/0.95 ± 1.65 at second follow-up (*p* = 0.742).

Eight patients had isolated strabismus sursoadductorius and showed improvement in the vertical deviation from 7.51 ± 4.98/7.71 ± 6.88 presurgical (near/distance) to 3.66 ± 3.07/6.86 ± 3.23 at first follow-up and 4.19 ± 3.67/6.86 ± 3.23 longterm in elevation in adduction (*p* = 0.167).

### Visual acuity

The mean age of the patients was 3.72 ± 1.36 years at time of the preoperative BCVA measurement, 3.77 ± 1.38 years at first follow-up and 4.88 ± 1.52 years at longterm follow-up.

BCVA was transformed to logMar for the statistical analysis and showed improvement in patients with esotropia from 0.20 ± 0.21/0.21 ± 0.22 (left/right) to 0.19 ± 0.22/0.18 ± 0.20 after 1 month and 0.20 ± 0.24/0.19 ± 0.25 at longterm follow-up (*p* = 0.392).

BCVA improved in patients with exotropia from 0.50 ± 0.29/0.42 ± 0.27 (left/right) to 0.31 ± 0.23/0.27 ± 0.21 at longterm follow-up (*p* = 0.55).

Preoperative BCVA in patients with isolated strabismus changed from 0.28 ± 0.36/0.25 ± 0.21 (left/right) before surgery to 0.08 ± 0.08/0.18 ± 0.18 at longterm follow-up (*p* = 0.80).

The Cardiff test was used for examining 12.90% of the children in this study while the remaining 87.10% were tested through the Lea test.

### Refractive error

The mean age of the patients was 3.72 ± 1.36 years and 4.88 ± 1.52 years at time of the preoperative and postoperative measurements, respectively. Mean refractive error was measured by the spherical equivalent and improved in the operated left eyes from 2.94 ± 2.20 to 2.09 ± 3.82 after surgery and from 2.84 ± 2.11 to 2.26 ± 3.07 in the operated right eyes in patients with esotropia (*p* = 0.312).

The refractive error in patients with exotropia changed from 0.09 ± 1.51/0.28 ± 1.44 (left/right) to 0.14 ± 1.63/0.25 ± 1.52 (left/right) (*p* = 0.689).

Patients with isolated strabismus sursoadductorius had a mean preoperative refractive error of 1.08 ± 4.45/0.75 ± 4.45 (left/right) which decreased to 0.17 ± 5.39/0.25 ± 5.19 (left/right) (*p* = 0.264).

### Stereopsis and Re-operations

The test for stereopsis was positive in 16,67% of patients preoperatively and in 38,46% of patients after surgery. The average age was 4.67 ± 0.93 years preoperatively and 5.41 ± 0.92 years after surgery. In 3.23% (*n* = 2) retreatment was necessary, one patient had combined esotropia with strabismus sursoadductorius and the other had combined exotropia with strabismus sursoadductorius. Both reoperations were conducted on the unilateral MOI after an average time span of 9.27 ± 7.74 months following the initial operation.

### Influencing factors

Change in AOD was significant in all three patient groups. Results also show improvement in other subcategories (elevation in adduction, BCVA and refractive error) but were not statistically significant. Multivariate logistic regression was performed to evaluate influencing factors of strabismus surgery. Table 6 shows the individual parameters and the results after the statistical analysis was conducted.

**Table 6:** Multivariate logistic regression analysis of parameters and successful outcome

Parameter	*P* value	Estimate ± Standard deviation	Odds ratio
Age at surgery	0.38	-0.412 ± 0.47	2.06
Gender	0.26	-0.32 ± 1.21	1.04
Type of strabismus	0.38	0.36 ± 0.41	1.44
Preoperative AOD	0.11	0.04 ± 0.04	1.04
Preoperative refractive error	0.11	0.43 ± 0.26	1.53
Preoperative visual acuity	0.88	0.37 ± 2.52	1.45

## Discussion

The purpose of this paper was to analyze the surgical outcome of strabismus surgery by using a retrospective data set. After running a multivariate logistic regression, there was no significant association between demographic parameters such as age at surgery or sex, preoperative AOD, refractive error, visual acuity or type of strabismus.

### Individual parameters and surgical success

The age at which strabismus surgery should be performed has been a subject for debate for many years. Early surgery can minimize harmful habits due to squinting and long lasting sensory changes but later surgery can lead to more accurate measurements and avoid unnecessary surgery [11–13] According to *Awadein *et al*.* [14] an age greater than 12 years old correlated with a worse surgical outcome. *Richard *et al*.* [12] showed that there was no significant difference in strabismus surgery outcome in the age group of 0–6 years. In our study, 50.00% of patients with a successful outcome were between 3 and 4 years old while 31.25% were younger and 1–2 years old. No significant difference was found in relation to age (*p* = 0.38).

*Abbsoglu *et al*.* [15] and *Kampanartsanyakorn *et al*.* [16] linked a larger preoperative AOD to a poorer surgical outcome. In this study, however, the preoperative AOD had no statistically significant influence on the surgical outcome (*p* = 0.38). A study from 2020 by *Waheeda-Azwa *et al*.* [17] reported similar findings to ours with a success rate of 81.60% that did not show a significant correlation to the preoperative AOD, with many patients having a large AOD. 13 out of 46 patients with a successful outcome in this study had a preoperative AOD of more than 20°, which could be explained by the young age group with possible congenital strabismus causes [17, 18].

### Change in the angle of deviation

Improvement in AOD was statistically significant in all patient groups. Patients with esotropia showed the greatest improvement (p < 0.001) followed by patients with isolated strabismus sursoadductorius (p = 0.037) and patients with exotropia (*p* = 0.004). Surgical success rate was 74.19% (*n* = 46) which correlates to findings of other similar studies [16, 19, 20]. Another study conducted by *Kumari *et al*.* [21] also reported a poorer success rate for exotropia in comparison with esotropia. A possible reason could be the inclination to postoperative drift in exotropia patients which results in a slightly poorer outcome.

Surgical management of exotropia includes the deliberate esotropic overcorrection as the initial postoperative result since it has been shown to provide good longterm outcomes [22–25]. But several studies have reported that initial overcorrection does not correlate with a more favorable longterm outcome [26–29]. Additionally, overcorrection in visually immature individuals, such as children aged 1–6 years, may be associated with consecutive esotropia, loss of stereopsis and amblyopia [24, 30]. It was also linked to a higher reoperation rate [28].

We did not aim for a postoperative overcorrection due to the reasons mentioned above and obtained satisfactory results with a success rate of 87.50% for patients with exotropia. The change between the first and longterm follow-up was not significant (*p* = 0.181).

### Change of the elevation in adduction

Improvement in elevation in adduction was shown in all three patient groups (*p* = 0.212). As *Stager *et al*.* [31] reported, more than 70% of patients with infantile esotropia and more than 30% of patients with intermittent exotropia have primary inferior oblique overaction. Overelevation in adduction has many possible causes that are difficult to correct with one surgical intervention. The study of *Stager *et al*.* [31] also reports that residual or recurrent over elevation in adduction is common.

### Change in visual acuity

Visual acuity did not show a significance in relation to the surgical success. The results of this study can be explained by the infantile patient group with the strabismus surgery taking place before visual maturity has fully occurred. *Kampanartsanyakorn *et al*.* [16] conducted a retrospective study of 304 patients to analyze the outcomes of horizontal strabismus surgery and found that preoperative visual acuity did not significantly influence the surgical success. The results of the study by *Ganguly *et al*.* [32] were similar and showed no significant change in visual acuity and binocular vision after strabismus surgery.

### Change in refractive error

The refractive error showed general improvement (*p* = 0.109) and was measured in the preoperative and long-term follow-up without measurements immediately after surgery. Several other studies conclude that strabismus surgery usually leads to short-term improvement regarding the refractive error with a long-term regression due to compensation or other postsurgical factors [33–36].

### Change in binocular vision

Obtaining the information on binocular and stereovision using the Lang test was only partially possible due to the young age of the examined patient group with a mean of four years. The data did not suffice for an in-depth analysis of binocular vision and stereovision. However, there is a trend toward postsurgical improvement with the percentage of a positive Lang test result rising from 16.67% to 38.46%. Children with a positive postsurgical Lang test result had a diagnosis of esotropia or strabismus sursoadductorius. The findings correlate with those of the study conducted by *Enz *et al*.* [37] who observed in their study a general improvement without a significant change in binocular vision and stereovision after surgery.

The critical time period for developing stereopsis starts at around 3 months after birth with further maturity until around 18 months of age [38–41]. Afterwards stereopsis gradually improves until the child is up to 3 years old [42]. Stereopsis was measured with the Lang test which is based on the random-dot technique and shows a cat, a star and a car representing disparities of 1200, 600 and 550 s of arc. The child then has to correctly name the objects [43]. A study by *Fawcett *et al*.* [44] investigated the critical period of susceptibility of stereopsis in patients with infantile esotropia and reported an onset at 2.4 months of age and the peak at 4.3 months of age. Other studies also reported the most rapid stereopsis development during the first 12 months after birth [39, 45]. Since we examined children aged 1–6 years in our study and do not have stereovision test results of all patients, we cannot make statements on the influence of strabismus on the development of stereopsis based on our data. We therefore suggest additional studies to further investigate the susceptibility of stereopsis that focus on the period of rapid maturity during the first year of life.

Certain limitations must be discussed as well. As a retrospective study we could only extract the data that were already collected and could not initiate further testing. But with this study we could show the outcome of strabismus surgery and influencing factors in an infantile age group. As a practical takeaway for the prospective surgical treatment of strabismus, the study shows that the overall success rate of strabismus surgery in infants is satisfactory at 74.19%.

## 5. Conclusion

In conclusion, the analysis showed that strabismus surgery has a good postoperative outcome and can be considered as a safe and effective treatment method for pediatric strabismus. Age, sex, type of strabismus, preoperative refractive error and visual acuity did not significantly influence the postoperative surgical success. Strabismus surgery in the infant showed a highly significant change in AOD with a low rate of retreatments but the best circumstances for surgical intervention are very individual and should be carefully evaluated.
